# The extravascular implantable defibrillator: a novel opportunity for the paediatric population

**DOI:** 10.1093/europace/euaf128

**Published:** 2025-06-26

**Authors:** Mauro Biffi, Andrea Quaranta, Alessandro Carecci, Luca Ragni, Andrea Donti, Cristian Martignani, Igor Diemberger, Andrea Angeletti, Matteo Ziacchi

**Affiliations:** IRCCS Azienda Ospedaliero Universitaria di Bologna, 40138 Bologna, Italy; Dipartimento di Scienze Mediche e Chirurgiche, Istituto di Cardiologia, Università di Bologna, 40138 Bologna, Italy; Dipartimento di Scienze Mediche e Chirurgiche, Istituto di Cardiologia, Università di Bologna, 40138 Bologna, Italy; IRCCS Azienda Ospedaliero Universitaria di Bologna, 40138 Bologna, Italy; IRCCS Azienda Ospedaliero Universitaria di Bologna, 40138 Bologna, Italy; IRCCS Azienda Ospedaliero Universitaria di Bologna, 40138 Bologna, Italy; Dipartimento di Scienze Mediche e Chirurgiche, Istituto di Cardiologia, Università di Bologna, 40138 Bologna, Italy; IRCCS Azienda Ospedaliero Universitaria di Bologna, 40138 Bologna, Italy; IRCCS Azienda Ospedaliero Universitaria di Bologna, 40138 Bologna, Italy

## Introduction

Sudden cardiac death due to malignant ventricular arrhythmias remains a significant threat, especially in paediatric patients with underlying heart disease. The implantable cardioverter-defibrillator (ICD) is the only established preventative therapy. However, current ICD systems—transvenous (TV) and subcutaneous (S-ICD)—are not always ideal for children due to their anatomical, physiological, and psychological characteristics.

A new extravascular ICD (EV ICD),^1^ which offers both defibrillation and anti-tachycardia pacing (ATP), combines the benefits of existing devices with a design more suited to younger patients. This case series reports the use of the EV ICD in seven paediatric patients, selected based on clinical need, anatomical feasibility, and limitations of traditional devices.

## Methods

Patients included in this series met Class I or IIa indications for ICD therapy for either primary or secondary prevention, as per international guidelines. Those requiring cardiac resynchronization therapy, bradycardia pacing, or with prior sternotomy were excluded. The evaluation process included clinical assessments, electrocardiogram, cardiac magnetic resonance imaging, and computed tomography scan when needed to guide EV ICD implantation. Specifically, lead placement was targeted to achieve an optimal positioning of the sensing electrodes (Ring1 and Ring2) in front of the right ventricle (RV) to ensure stable R-wave sensing while avoiding atrial signal oversensing, which can cause inappropriate detection and therapy delivery.^2^

## Results

### Patient characteristics

Seven patients, mean age 13.2 years (11–16) and mean body mass index of 17.9 ± 1.6 kg/m², underwent implantation from May 2024 to March 2025. Left ventricular ejection fraction averaged 50%, and the average NYHA (New York Heart Association) class was 1.3. Implantable cardioverter-defibrillator indication was: primary prevention in five patients (71.4%) and secondary prevention in two patients (28.6%).

Underlying cardiomyopathies: high-risk non-obstructive hypertrophic cardiomyopathy (3), Duchenne-related dilated cardiomyopathy (2), Hypokinetic dilated cardiomyopathy (1), Arrhythmogenic cardiomyopathy (ACM) associated to desmoplakin mutation (1).

All patients had scarred area/layers at the MR (Magnetic Resonance) scan, none was on anticoagulation. One patient was ineligible for a wearable defibrillator due to small body size, one failed the S-ICD screening test, four expressed concerns about the impact of S-ICD, favouring the EV ICD.

### Procedural outcomes

Implants occurred either in general anaesthesia (two patients) or deep sedation with local analgesia (five patients), depending on patient needs and parental preference.

**Figure 1:**
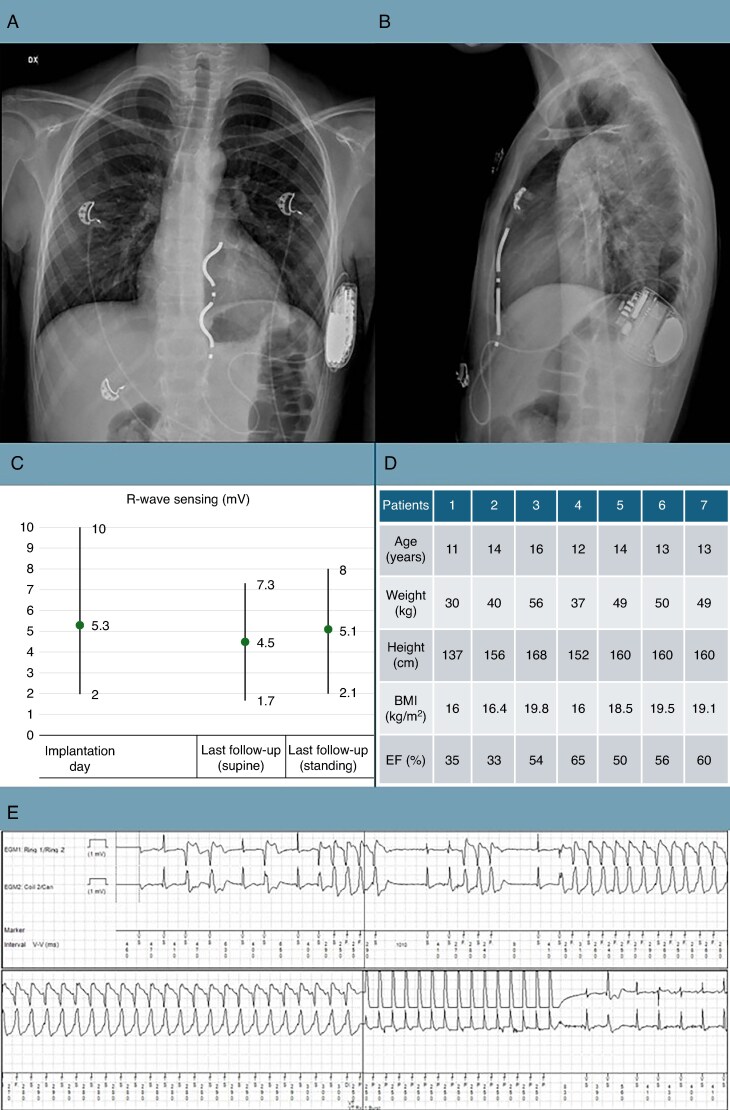
(*A*, *B*) Implant in a 12 years old boy with hypertrophic cardiomyopathy. See Ring 1 and 2 in front of the RV, the abdominal loop, and the intermuscular placement. (*C*) Trend of mean R-wave amplitude, from implantation to last follow-up. (*D*) Body habit and ventricular function. (*E*) 16 years old girl with desmoplakin-related arrhythmogenic cardiomyopathy, cluster of NSVTs (Non Sustained Ventricular Tachycardia) followed by VT terminated by ATP. ATP, anti-tachycardia pacing; RV, right ventricle.

All leads were successfully tunnelled at the first attempt. The distance between sternum and pericardium was <5 mm in all patients. Leads were placed along the left sternal border or slightly leftward with a subcutaneous abdominal loop to accommodate body growth (*Figure 1A* and *B*) and to avoid steep lead angles, whereas the device was placed intermuscular for improved tolerance while maintaining defibrillation efficacy.^3^ The average R-wave amplitude was 5.3 ± 2 mV in Ring1–Ring 2 configuration, the P wave was never detectable in any patient. Defibrillation testing during implantation demonstrated a 100% success rate: five patients were successfully defibrillated with 15 J, one required 20 J and one required 30 J (average 18 ± 5 J).

Median procedure duration (from sterile preparation to patient recovery) was 97 ± 14 min, with fluoroscopy averaging 2 min and 40 s.

### Follow-up and complications

At a mean follow-up of 9 ± 6 months, device function remained stable in all patients. One patient underwent heart transplantation after 10 months. The EV ICD lead was easily removed during surgery without adhesions. Four patients resumed non-competitive sports: swimming, five-a-side football, trekking.

### Device performance at follow-up

R-wave amplitude in Ring1–Ring2 configuration was 4.6 ± 2 mV. Device detection was effective with no inappropriate detections or therapy delivery. The patient with ACM experienced several NSVTs, followed by two sustained VTs (Ventricular Tachycardia) respectively at 214 b.p.m. and 240 b.p.m. at 3 and 7 months follow-up, both successfully terminated by ATP at the first attempt (*Figure 1E*). Estimated longevity was 10.6 ± 0.4 years (range 10–11).

## Discussion

This case series highlights the promising role of the EV ICD in the paediatric population. While children were excluded from the PIVOTAL trial, the device offers a safe and effective option when TV or S-ICDs are not ideal. Key advantages for paediatric use include: ATP functionality, crucial for terminating monomorphic VT episodes shockless; long battery life, reducing the number of replacements and associated complications; extravascular placement, which avoids vascular access and reduces the risk of infection or lead-related issues.

These benefits meet the needs of younger patients who face long-term device dependency. Traditional TV ICDs offer ATP, but require intravascular leads, which increase the risk of endovascular infections and mechanical failure over time. Subcutaneous implantable cardioverter-defibrillator is on the other hand bulkier, lacks ATP, and has shorter battery life.

The EV ICD bridges these gaps in patients facing body growth. The guidance of CT/MR scan enabled an optimized lead positioning to obtain reliable R-wave amplitudes while minimizing the detection of the P wave and of non-ventricular signals. In this way, we were able to rely solely on the Ring1–Ring2 sensing vector, thereby avoiding those sensing issues which exposed to inappropriate shock delivery in the PIVOTAL trial.^1,2^ Indeed, the mean R-wave amplitude is greater than the recently reported ENLIGTHEN registry,^4^ possibly owing to the narrow space between the sternum and the pericardium observed our patients, and to the targeted lead placement in front of the RV.

Intermuscular generator placement further improved cosmetic outcomes and comfort, factors highly relevant in paediatric care. Lead and device performance at follow-up looks promising in our patients, when considering that complications’ rate of transvenous ICDs in this age subgroup may reach 8–10% in the first year.^5^

Although more long-term data on lead performance and extractability are awaited, recently reported data hint at rather safe lead extraction procedures,^6^ which is relevant for young patients.

## Conclusion

The EV ICD represents a valuable additional therapy for paediatric patients at risk of sudden cardiac death, particularly when other devices are unsuitable due to anatomical, clinical, or psychological considerations. It provides the combined advantages of effective defibrillation, ATP, extravascular lead placement, and small size—all critical for younger patients with long life expectancy.

Our experience shows that, with careful planning and imaging-guided implantation, the EV ICD offers reliable arrhythmia management, good procedural safety, and excellent patient acceptance. This technology has the potential to significantly improve care for paediatric patients requiring defibrillator therapy.

## Data Availability

The data underlying this article will be shared on reasonable request to the corresponding author.
